# Pyrolyzed biomass-derived nanoparticles: a review of surface chemistry, contaminant mobility, and future research avenues to fill the gaps

**DOI:** 10.1007/s42773-022-00152-3

**Published:** 2022-06-02

**Authors:** Logan Swaren, Salman Safari, Kurt O. Konhauser, Daniel S. Alessi

**Affiliations:** Department of Earth and Atmospheric Sciences, University of Alberta, 3-16 Earth Sciences Building, Edmonton, AB T6G 2E3 Canada

**Keywords:** Nanoparticles, Surface chemistry, Activated carbon, Biochar

## Abstract

Nanoparticles are abundant in the subsurface, soil, streams, and water bodies, and are often a critical control on elemental speciation, transport and cycling in the natural environment. This review provides an overview of pyrolyzed biomass-derived nanoparticles (PBNPs), their surface properties and reactivity towards aqueous species. We focus specifically on biochar-derived nanoparticles and activated carbon-derived nanoparticles which fall under our classification of PBNPs. Activated carbon-iron (nano)composites are included in some instances where there are significant gaps in literature because of their environmental relevance. Increased use of activated carbon, along with a resurgence in the manufacture and application of biochar for water treatment and soil amendment, has generated significant concerns about the mobility and toxicity of PBNPs derived from the bulk material in environmental applications. Recent examples are discussed to highlight current progress in understanding the influence of PBNPs on contaminant transport, followed by a critical discussion of gaps and future research directions.

**Graphical Abstract:**

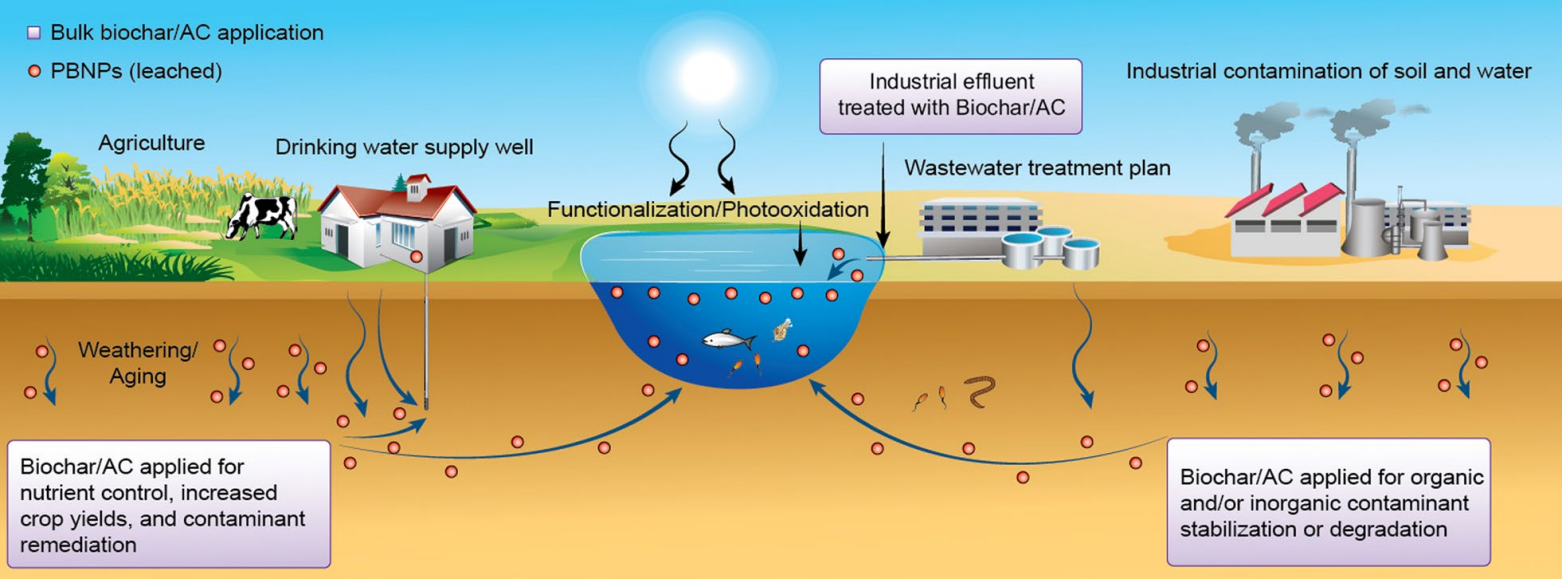

## Highlights

- The surface functionality of pyrolyzed biomass-derived nanoparticles (PBNPs), described here as those derived from bulk biochar and activated carbon, differs from parent material.
- The literature on PBNPs derived from biochar is growing, unlike that of activated carbon derived PBNPs.
- There is a need for better understanding of co-transport of contaminants, photochemistry, and toxicity of PBNPs.

## Introduction

Pyrolyzed biomass-derived nanoparticles (PBNPs) are those particles limited in all three dimensions to < 100 nm (for more detail on nanoscale definitions see Hochella et al. 2008). There are various forms of PBNPs, but this review focuses on the implications of the surface reactivity and contaminant interactions of PBNPs leached from bulk parent materials that are widely used in environmental applications, i.e., activated carbon (AC) and biochar. Studies on purpose-produced biochar nanoparticles and activated carbon (AC) nanoparticles, activated carbon derivatives, and activated carbon-iron composites are included where possible for comparison. Activated carbon is a common industrial product that has been studied extensively, whereas biochar can be created naturally through wildfires (Scott et al. 2014) or manufactured for research or commercial activities, with most research on biochar properties occurring only over the past decade (Wu et al. 2019). The most distinct difference between AC and bulk biochar is the “activation” process in the pyrolysis of AC, whether it is chemical or physical (detailed in Sect. 2).

There are thousands of published papers investigating the interactions of AC or biochar with various inorganic and organic contaminants, as well as investigations of the influence of production conditions and the use of various biomass feedstocks on the physicochemical properties of the resulting carbonaceous products (Ahmad et al. 2014; Mohan et al. 2014; Gonzalez-Garcia 2018; Heidarinejad et al. 2020; Wang et al. 2020a, b; Mukhopadhyay et al. 2021). Recently, engineered nanoparticles (ENPs), intentionally or unintentionally released, have come under investigation due to their influence on contaminant transport and potential toxicity in the environment (Abbas et al. 2020). Unfortunately, these previous reviews focus solely on ENPs, such as fullerenes, nanotubes, and metal-oxides, as opposed to PBNPs leached from bulk biomass-derived adsorbents. Recent reviews, such as Ramanayaka et al. (2020a), provide a thorough review of nanobiochar, its synthesis, surface chemistry, and environmental applications such as soil amendment, but lack in discussion of the long-term persistence of PBNPs in the environment. This gap in understanding is likely because the scientific literature on PBNPs is just emerging and no long-term studies have been conducted. Nonetheless, it is imperative that this gap is recognised, as literature to date does not investigate the production, fate, and interactions of PBNPs derived from bulk AC or biochar applications in nature.

There are various pathways in which PBNPs can be naturally derived from bulk parent applications. Some of these mechanical processes have been identified through laboratory studies, such as pore collapse and the fracturing of biomass during pyrolysis and breakage due to mechanical grinding (Liu et al. 2018; Ramanayaka et al. 2020a). These processes may occur through processes such as freeze–thaw cycles and other physical abrasions. Additionally, there are investigations that isolated nanoparticles by suspension and removed the macro-fraction of biochar via centrifugation (Ramanayaka et al. 2020b). Although there is no direct comparison of reactivities between PBNPs purposefully produced (e.g., ball-milled) and those leached after bulk material application and derived through natural processes, it is safe to assume that mechanical breakage is comparable to abrasion and other physical stresses imposed in the environment. Recently, Zhong et al. (2020) investigated the weathering and aging effects on bulk biochar structure and surface chemistry used in soil amendment, which may additionally include degradation induced by microbial communities. To our knowledge, there has been no review of the impacts of PBNPs produced and released in the environment from biochar and activated carbon used in remediation, soil amendment, and wastewater treatment applications.

## Activated carbon

Activated carbon (AC) refers to a variety of synthesized, amorphous, carbonaceous materials that have higher surface area and porosity than their source material. Because of the surface and textural characteristics of AC, it has been utilized in contaminant removal and various other applications discussed below (Uner et al. 2019). The properties of AC are largely dependent on the precursor material, which is usually any form of biomass with a high carbon content (Danish et al. 2013). A wide variety of precursor materials have been investigated that often include fast growing, lignocellulosic biomaterials associated with agricultural and commercial wastes (see Yahya et al. 2015; Gonzalez-Garcia 2018; Heidarinejad et al. 2020 and references within). In addition to the precursor material, the method of carbonization and activation plays a fundamental role in determining AC properties.

There are two processes for the preparation of AC : physical activation and chemical activation. Physical activation requires pyrolysis, referred to as carbonization, of the precursor material prior to activation (Bouchelta et al. 2008; Yahya et al. 2015). Carbonization typically occurs at temperatures between 400 and 1000 °C under inert, O_2_-free atmospheric conditions to maximize the carbon concentration of the resulting charcoal—referred to as the “carbonaceous skeleton” (Lewis 1982; Gonzalez-Garcia 2018; Heidarinejad et al. 2020). Investigators must carefully choose or optimize carbonization parameters such as temperature, heating rate, type and injection rate of inert gas, and residence time because they all can influence the resulting AC properties (Daud et al. 2000; Lua et al. 2006). The second step in physical activation following carbonization is gasification using an oxidizing agent, such as CO_2_, or steam to produce higher porosity (Molina-Sabio and Rodriguez-Reinoso 2004). Physical activation can also be achieved through a one-step procedure where a CO_2_ or N_2_ atmosphere is present during the pyrolysis process.

Chemical activation is considered more advantageous in the production of ACs because carbonization and activation are achieved in a single step, it typically requires lower temperatures, and there is more control over resulting porosity (Tay et al. 2009; Laksaci et al. 2017). Activation using this method occurs through reactions with chemical reagents including, but not limited to, ZnCl_2_, H_2_SO_4_, KOH, NaOH, HCl, and K_2_CO_3_ (see Yahya et al. 2015; Gonzalez-Garcia 2018; Heidarinejad et al. 2020 and references within for more extensive lists of activating reagents). The mixing of precursor biomaterial and chemical reagents is performed either through impregnation via submersion in solution followed by filtering, or through physical mixing in the absence of water (Heidarinejad et al. 2020). It has been demonstrated that the method of chemical activation and varying pre- and post-pyrolysis parameters (e.g., impregnation ratio, temperature, and final washing solution) heavily influence the distribution of porosity, size of pores, and surface area of AC (Heidarinejad et al. 2020). Chemical activation results in greater porosity when compared to physical activation because the activating agent reacts with the carbon matrices of the material, leading to the removal of gas products to produce a porous structure (Molina-Sabio and Rodriguez-Reinoso 2004). For example, many researchers have found a positive correlation between activating agent dosage and surface porosity due to evaporation of the agent during activation (Vicinisvarri et al. 2014; Yakout et al. 2016). Some parameters to be considered when preparing AC with homogenous and controlled porosity include a strict selection of the precursor biomass and a well-understood activation agent. Molina-Sabio and Rodriguez-Reinoso (2004) provided a thorough investigation of the effect of various activating agents on the resulting porosity of AC. Given the wide array of precursor materials and numerous activation techniques and parameters that can be adjusted, the properties of ACs have been extensively studied for a wide array of applications.

PBNPs may be formed through several mechanisms during AC production and following its application. The mechanical reduction of bulk AC to PBNPs through ball-milling has been suggested to be more cost-effective and scalable than chemical processes such as solution-based precipitation (Gao et al. 2015). Following environmental applications, AC may experience mechanical weathering, such as freeze–thaw cycles and wet–dry cycles, in addition to various biochemical processes, such as biological oxidation, leading to the production of PBNPs.

### Surface functionality

The surface reactivity activated carbon nanoparticles (CNPs) are distinct from their parent biomass. Unfortunately, only a limited number of studies have investigated CNPs (herein referred to as PBNPs) as distinct entities from their parent sorbent. From an environmental perspective, most PBNP reactivity is extrapolated from investigations on the parent sorbents or studies on synthesized PBNPs for various other technological applications (e.g., optical properties). Fourier transform infrared (FTIR) spectroscopy indicates that following activation, PBNPs typically demonstrate a decrease in oxygen containing functional groups and an increase in C=C vibrations attributed to an abundance of aromatic structures on the surface (Friedel and Hofer 1970; Koseoglu and Akmil-Basar 2015; Saygili and Guzel 2016; Jain et al. 2018). Both AC and PBNPs exhibit broad band stretching around 3400 cm^−1^ which are attributed to phenolic –OH groups (Jain et al. 2018). Additionally, many researchers have identified the presence of C–O stretching (Saygili and Guzel 2016), C–H stretching of alkane carbon (Yogesh et al. 2017), and C=O conjugated vibrations (Pouretedal and Sadegh 2014). Jain et al. (2018) identified the presence of –OCH_3_ groups likely corresponding to lignin groups associated with sunflower head waste PBNPs.

The surface reactivity of PBNPs is largely controlled by activation conditions, making their production largely specialized to the intended application (Heidarinejad et al. 2020). Hu et al. (2009) investigated the surface properties of PBNPs resulting from graphite suspended in various reagents including diamine hydrate, diethanolamine, and polyethylene glycol that all produced an abundance of carboxylate surface functional groups. When taking those same PBNPs and boiling them in perchloric acid, the surface functional groups transformed to primarily methyl groups (Hu et al. 2009). Moreover, nitric acid oxidation of PBNPs is well documented to produce hydroxyl and carboxyl surface functional groups (Ray et al. 2009; Yogesh et al. 2017).

In addition to surface functional group modification, physical characteristics have been found to change considerably during the production of PBNPs from bulk AC, such as specific surface area and hydrophobicity. Baheti et al. (2015) found an increase of 55% in AC surface area following 3 h of ball milling (Table 1). By contrast, Gao et al. (2015) identified a large decline in specific surface area from 895.3 to 94.3 m^2^ g^−1^ after 4 h of ball milling (Table 1). They attributed this loss of surface area to a collapse of the pore spaces that was identified by a tenfold decrease in pore volume (Gao et al. 2015).

**Table 1:** Summary of specific surface area properties of bulk AC and derived PBNPs

Bulk AC/PBNPs	Specific surface area (m^2^ g^−1^)	References
Acrylic fibrous waste AC	278	Baheti et al. (2015)
*PBNP* (via ball-milling)	432	
Powdered AC	895	Gao et al. (2015)
*PBNP* (AC-ZVI nanocomposite via ball-milling)	100	
*PBNP* (ball milled AC)	94	
*PBNP* (synthesized Fe_3_O_4_-AC nanocomposite)	104	Ece (2021)

Previous investigations have found that decreasing surface functional group densities of pyrolyzed material results in more hydrophobic behaviour due to less binding capacity to water (Goncalves et al. 2010; Weber and Quicker 2018). A decrease in surface functional groups is observed with increasing pyrolysis temperature of parent AC (Goncalves et al. 2010) and some ENPs exhibit super-hydrophobic properties (Betar et al. 2021), although no trends in hydrophobicity have been reported with respect to AC-derived PBNPs to our knowledge. Moreover, there is a lack of literature investigating the differences in the zeta potential and carbon content of parent AC and PBNPs derived from this AC. If extrapolating from biochar nanoparticle investigations, one would expect that zeta potential reaches more negative values and carbon content decreases (Oleszczuk et al. 2016; Chausali et al. 2021), although experiments need to be conducted to confirm this conjecture. In the limited number of studies that quantify the physical properties of both parent AC and daughter PBNPs, there is not a clear trend between initial pore volume of the AC and resulting PBNPs. To better predict the physical properties of PBNPs, emphasis should be placed on developing a better understanding of the impacts of production procedures.

### Influence on contaminant removal

#### Inorganic contaminants

There is an abundance of literature covering the use of ENPs, including carbon-based ENPs (e.g. graphene oxide, carbon nanotubes, fullerenes) on inorganic contaminant removal from aqueous solutions, typically focused on recovery of heavy metals from industrial wastewater (Maitlo et al. 2019; Yang et al. 2019a, b; Lal et al. 2020). There is limited research on utilizing PBNPs for heavy metal remediation, nor are there reviews on the topic. To adequately investigate the interactions and predict the fate of PBNPs in the environment as well as their suitability as inorganic contaminant remedial agents, we focus on discussing a variety of PBNPs, including binary Fe–C adsorbents. The production of binary Fe–C adsorbents is far more studied than AC-derived PBNPs, and in most binary sorbents zero-valent iron (ZVI) is employed as an electron donor to reduce potentially toxic contaminants in the environment (Hoch et al. 2008). We discuss Fe-composites in this review, as Fe is common in the environment, especially in comparison to other binary AC sorbents reported in the literature (e.g., Ti, Co, Ag). In these adsorbents the C, commonly AC, acts as an electron shuttle.

Pyrzynska and Bystrzejewski (2010) synthesized carbon-encapsulated nanoparticles (PBNPs) consisting of a Fe(0) core with an ~ 1 nm carbon coating for the removal of Co(II) and Cu(II) from aqueous solution. They found that the PBNPs had a higher surface charge density compared to their AC parent material (4.74 and 2.25 C m^−2^, respectively). Co(II) removal by PBNPs was nearly 100% in ultrapure water and decreased to ~ 86% in 0.5 mol L^−1^ NaCl, whereas bulk AC removed ~ 60% and ~ 52%, respectively (Pyrzynska and Bystrzejewski 2010). Sorption of Cu(II) followed a similar trend with PBNPs removing ~ 100% and ~ 86% in ultrapure water and 0.5 mol L^−1^ NaCl, respectively (Pyrzynska and Bystrzejewski 2010). The decrease in adsorption with increasing NaCl can be accounted for by monovalent cation adsorption (Alessi et al. 2010), where surface sites responsible for binding divalent cations compete with an increasing abundance of Na^+^ in solution. Understanding the adsorption capacity of PBNPs across varying salinities is crucial in attempts to assess the mobility of PBNPs in aqueous environments. Given the higher cation adsorption capacity at lower salinities, PBNPs could act as a transport mechanism of adsorbed contaminants migrating from low salinity environments such as freshwater streams, to saline environments such as estuaries or aquifers. When considering AC as a remediation technique, the regional hydro(geo)logy must be considered to understand how PBNPs leached from AC may migrate in the environment.

**Fig. 1:**
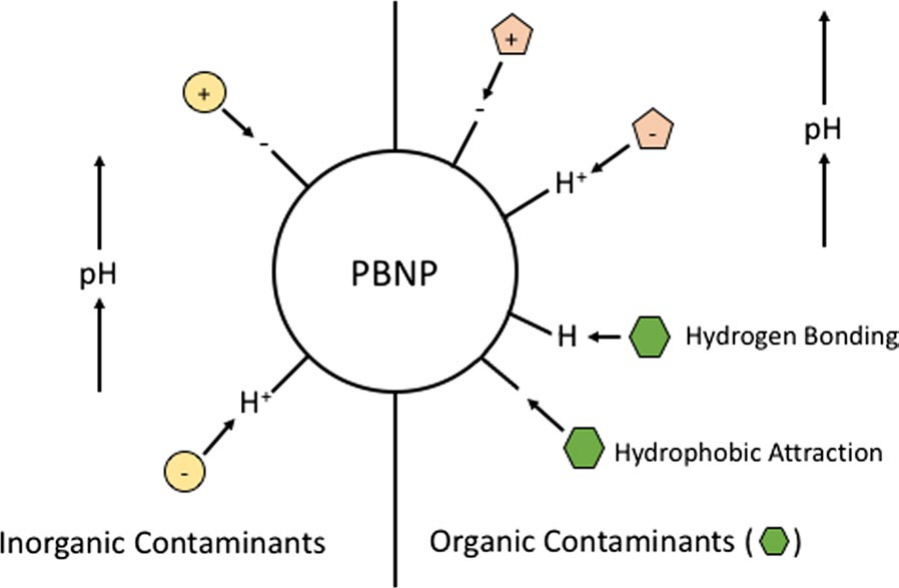
Schematic diagram of the surface of PBNPs as a function of increasing solution pH, demonstrating mechanisms of contaminant adsorption

Recently, Jain et al. (2018) compared the efficiency of magnetite (Fe_3_O_4_) and sunflower head waste derived Fe_3_O_4_-AC nanoparticle composites (PBNPs) for removal of Cr(VI), Cu(II) and Cd(II) from solution. Both systems demonstrated nearly instantaneous adsorption of all metals investigated, but the adsorption capacity of the PBNPs outmatched that of Fe_3_O_4_ nanoparticles alone (Jain et al. 2018). The PBNPs were capable of > 90% removal of Cr(VI) and Cd(II) and > 80% removal of Cu(II), whereas the Fe_3_O_4_ nanoparticles removed ~ 60%, ~ 10%, and ~ 25%, respectively. The maximum removal efficiency was determined to be at pH 2 for Cr(VI) and pH 6 for both Cu(II) and Cd(II). The difference in maximum adsorption is a result of contaminant speciation, where Cr(VI) is present as an oxyanion and therefore adsorbs more efficiently at low pH when the surface functional groups are protonated (Fig. 1). The opposite is true for Cd(II) and Cu(II), where maximum adsorption occurs at higher pH as functional groups become negatively charged following deprotonation (Fig. 1). The effect of PBNP embedding on the efficiency of water treatment membranes in removing Cu(II) from solution depends on the PBNP loading (Hosseini et al. 2018). Hosseini et al. (2018) found that bamboo-derived PBNPs embedded in polyethersulfone membranes reduced the effluent concentration of 20 mg L^−1^ Cu(II) contaminated water by 80% with a 0.05 (wt%) dosage of PBNPs and > 95% at a 0.5 (wt%) dosage.

The influence of PBNPs on the mobility of inorganic contaminants in aqueous environments has received little attention in recent years. It is probable that PBNPs behave like previously studied ENPs with regards to mobility in soils and aquifers and co-transport of inorganic contaminants. There are various factors that can influence the co-transport of PBNPs and inorganic contaminants, such as PBNP surface chemistry, physical properties, matrix properties, contaminant type, and solution properties (Jiang et al. 2018). The mobility of contaminants is contaminant specific due to differences in adsorption affinity to the surrounding matrix, speciation of the inorganic contaminant in solution, and potential contaminant influences on PBNP aggregation (Ling et al. 2021). Understanding these factors in aqueous environments is pivotal in understanding the role of PBNPs in the transport and fate of inorganic contaminants. Our understanding is hampered by the absence of laboratory-based experiments providing the fundamental thermodynamic framework to predict particle behaviors in complex natural environments.

#### Organic contaminants

Various types of PBNPs have been utilized for the remediation of organic contaminants. Fan et al. (2017) provide recent insights into in-situ AC-based organic contaminant remediation strategies, including those based on PBNPs, including Carbo-Iron^®^ colloids (CICs), colloidal AC, and various other zero valent iron (ZVI) impregnated ACs (Table 2). CICs consist of nano-ZVI clusters that are embedded in colloidal (~ 1 µm) AC (Bleyl et al. 2012). Moreover, many studies have investigated the capacity of these composites for the dechloronation of hazardous chlorinated hydrocarbons such as tri- and tetrachloroethene, TCE and PCE, respectively (see Table 2 for a summary of studies), building on earlier studies that proved the efficacy of ZVI in hydrocarbon dechlorination (Matheson and Tratnyek 1994; Alessi and Li 2001).

**Table 2:** An overview of recent Fe/AC nanoparticle studies on the adsorption of organic contaminants

PBNPs	Contaminants	References
CIC	CE	Czinnerova et al. (2020)
	PCE	Weil et al. (2019), Vogel et al. (2018), Mackenzie et al. (2016)
Fe_3_O_4_-AC nanocomposite	Benzene and Toluene	Ece (2021)
Sulfur treated CIC	PCE	Vogel et al. (2019)
MAC nanoparticles	MB and RR	Abuzerr et al. (2017)
nZVI + Colloidal AC	DDT	Kopinke et al. (2020)
Ball-milled ZVI + AC	TCE	Guan et al. (2020), Gao et al. (2015)
CNC-nZVI	MO	Bossa et al. (2017)

*CIC* Carbo-Iron colloids, *CNC* cellulose nanocrystal, *CE* chlorinated ethenes, *MAC* magnetic activated carbon, *MB* methylene blue, *MO* methyl orange, *nZVI* nanoscale zero-valent iron, *RR* reactive red

Mackenzie et al. (2016) conducted the first field scale investigation of the capability of CICs to degrade a PCE contaminated site in Germany. The study found that after one injection of CICs into the subsurface there was a close and immediate correlation between decreasing PCE (~ 24 mg L^−1^ to ~ 2 mg L^−1^) and increasing ethane (Mackenzie et al. 2016). Following a second injection, the PCE concentration was reduced from 19 mg L^−1^ to nearly zero, and only increased to 1.5 mg L^−1^ after 200 days (Mackenzie et al. 2016). Interestingly, Vogel et al. (2018) found that sulfidation of CICs at as low as a 0.004 S/Fe molar ratio greatly increased their resistance to anaerobic corrosion in the subsurface. Compared to untreated CICs that lost 70% of their reduction capacity within 15 days, the sulfur treated CICs only experienced a 20–25% loss in reduction capacity after 160 days (Vogel et al. 2018). In addition to extended lifetime, the sulfur treated CICs dechloronation efficiency increased by approximately 3 times to achieve a 98% removal efficiency of 200 µM PCE (Vogel et al. 2018). Although CICs fall slightly outside the operation definition of nanoparticles, they are considerably smaller than bulk AC and are certainly more reflective of PBNP properties.

**Fig. 2:**
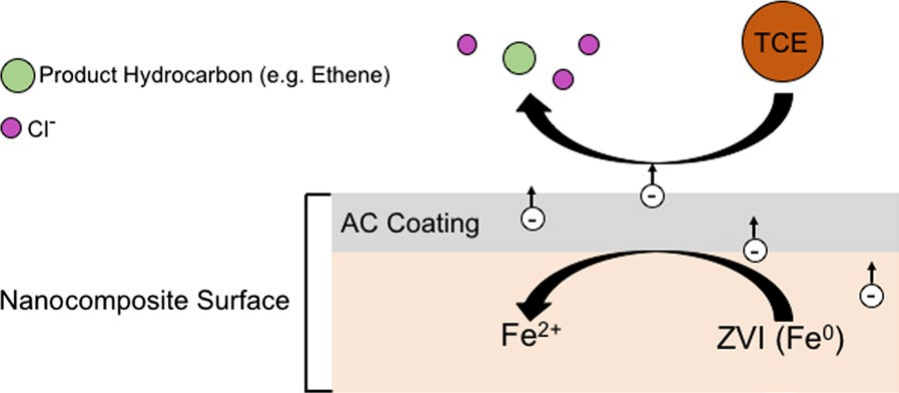
Schematic diagram of trichloroethene (TCE) degradation via electron shuttling by a generalized AC-ZVI nanocomposite PBNP

The mechanical reduction of macro-scale adsorbents to nanoparticles via ball-milling has been suggested to be more cost-efficient and scalable than other methods such as gas-phase reduction or solution-based precipitation (Gao et al. 2015). In a study comparing the reactivities of admixtures of ZVI + AC and ball-milled ZVI-AC nanocomposites toward TCE, Gao et al. (2015) found that dechlorination occurs immediately in both systems but decreases rapidly with time in the ZVI + AC mixture. The authors showed that this is due to the eventual separation of ZVI and AC in solution because of contrasting densities resulting in an inability for electrons to transfer from the ZVI to the AC during TCE reduction. After extended contact time, the TCE can still be reduced by the ZVI-AC nanocomposites because the ZVI is embedded in the AC structure. Guan et al. (2020) also investigated the influence of AC loading on ZVI following ball-milling with respect to TCE degradation and unproductive ZVI reactivity with environmental H_2_O/H^+^. They found that two factors must be balanced for optimal performance: the presence of a thick enough carbon coating (e.g., 10% carbon loading) to protect the ZVI particles from reacting with environmental H_2_O/H^+^ (which decreases the lifetime of the particles), and a thin enough coating (e.g., 1% carbon loading) to allow for rapid degradation of TCE via electron shuttling from ZVI to the AC (Fig. 2) (Guan et al. 2020). However, Guan et al. (2020) investigated micro-ZVI-AC composites, the electron transfer mechanism for TCE degradation would occur at both macro- and nano-scale in the presence of PBNPs and ZVI.

The study of PBNPs and ionic organic pollutants, such as dyes, has been largely limited to PBNP-magnetic composites for the purpose of dye collection and recycling. The interaction between the cationic and anionic organic pollutants (methylene blue and reactive red, respectively) and magnetic activated carbon (MAC) nanoparticles is principally a function of pH (Abuzerr et al. 2017). Those authors observed that methylene blue was optimally removed from aqueous solution at pH 10, whereas reactive red was most efficiently removed at pH 5.5. The PBNP adsorption capacity remained unchanged following 10 adsorption/desorption cycles. The reasons that dye adsorption is pH-dependent remain unstudied for PBNPs, although it is likely due to the reversible deprotonation of acidic surface functional groups (e.g., carboxyl) which have been previously identified in bulk AC (Fig. 1) (Laksaci et al. 2017). Combining adsorption and column experiments, Bossa et al. (2017) found that nanoscale ZVI (nZVI) synthesized by cellulose nanocrystals (CNC-nZVI) was capable of 25% more methyl orange removal than nZVI alone. Moreover, CNC-nZVI demonstrated stable colloidal properties optimal for transport in porous media, making it a suitable candidate for pumping and treating environmental remediation (Bossa et al. 2017).

The physiochemical properties of the organic contaminant of interest such as hydrophobicity and molecule size play an important role on the effectiveness of PBNPs as a sorbent. High carbon content, characteristic of lignin-derived materials, results in a small number of polar functional groups, causing the particles to be hydrophobic and separate from water (Senesi 1992). Gao et al. (2015) found that ball-milled ZVI-AC nanocomposites were hydrophobic, making them ideal for the remediation of similarly hydrophobic contaminants such as TCE. The molecular size of the PBNP and the nature of the target organic contaminant directly correlate to the extent of the van der Waals forces acting on subsequent adsorption; therefore, molecular size must be taken into account when determining the optimal surface area of PBNP for the remediation of large organic molecules that are contaminants in water (Senesi 1992).

Due to their toxicity to aquatic life and adverse effects on humans (e.g., carcinogenic), the contamination of water by trace concentrations of pharmaceuticals is an emerging environmental and health concern (Mansour et al. 2018). The adsorption of the commonly applied antibiotics, Amoxicillin, Cephalexin, Tetracycline and Penicillin G, to PBNPs produced from vine wood revealed that as little as 0.4 g L^−1^ adsorbent could remove 20 mg L^−1^ antibiotics from an aqueous solution (Pouretedal and Sadegh 2014). Additionally, they found that the PBNPs could be reused by desorption of the antibiotics in 5 (w/w%) NaOH in 4 h (Pouretedal and Sadegh 2014). Therefore, wastewater treatment systems employing bulk AC for the removal of pharmaceuticals need to consider the potential for PBNPs leaching from the treatment pipeline and the persistence of those PBNPs in the environment.

## Biochar-derived nanoparticles

Carbon-rich materials produced by the pyrolysis of biomass, such as wood or manure, under limited to no oxygen are collectively referred to as biochar (Ahmad et al. 2014; Lehmann and Joseph 2015). The high carbon content, large specific surface area, and the presence of reactive chemical functional groups have made biochar a unique sorbent to remove both organic and inorganic pollutants from aqueous systems (Xie et al. 2015; Oliveira et al. 2017).

Recent studies have found that the micropores of biochars can release carbonaceous nanoparticles (PBNPs) and colloids when submerged in water (Qu et al. 2016; Liu et al. 2018). The increasing applications of bulk biochars in soil remediation, soil amendment and water treatment may lead to the leaching of this fraction of biochar into groundwater, surface runoff, and ultimately into waterways and the oceans. The interactions of PBNPs with metals, organic compounds and other natural compounds in aqueous systems require further study, particularly considering the increasing use of biochars in water treatment and agricultural applications.

Pyrolyzed biomass nanoparticles can be produced through several mechanisms during biochar production and after its application. During production, pore collapse and matrix fracturing contribute to the generation of PBNPs (Liu et al. 2018). Once applied to soil systems, biochars may experience natural physical weathering, such as freeze–thaw cycles and wet–dry cycles as well as chemical and microbiological degradation, leading to disintegration into nanoscale particles (Lian and Xing 2017). Initial toxicological research shows that these PBNPs themselves are not acutely toxic toward the model organism *Daphnia magna* (water flea) (Safari et al. 2019). However, due to the distinct physicochemical characteristics and the unique colloidal behavior in the natural environment, they may facilitate or hinder the transport of contaminants in the environment under different physiochemical conditions thereby influencing toxicity, a subject that has garnered more attention in the past several years (Wang et al. 2019; Fang et al. 2020; Meng et al. 2021).

### Surface functionality

**Fig. 3:**
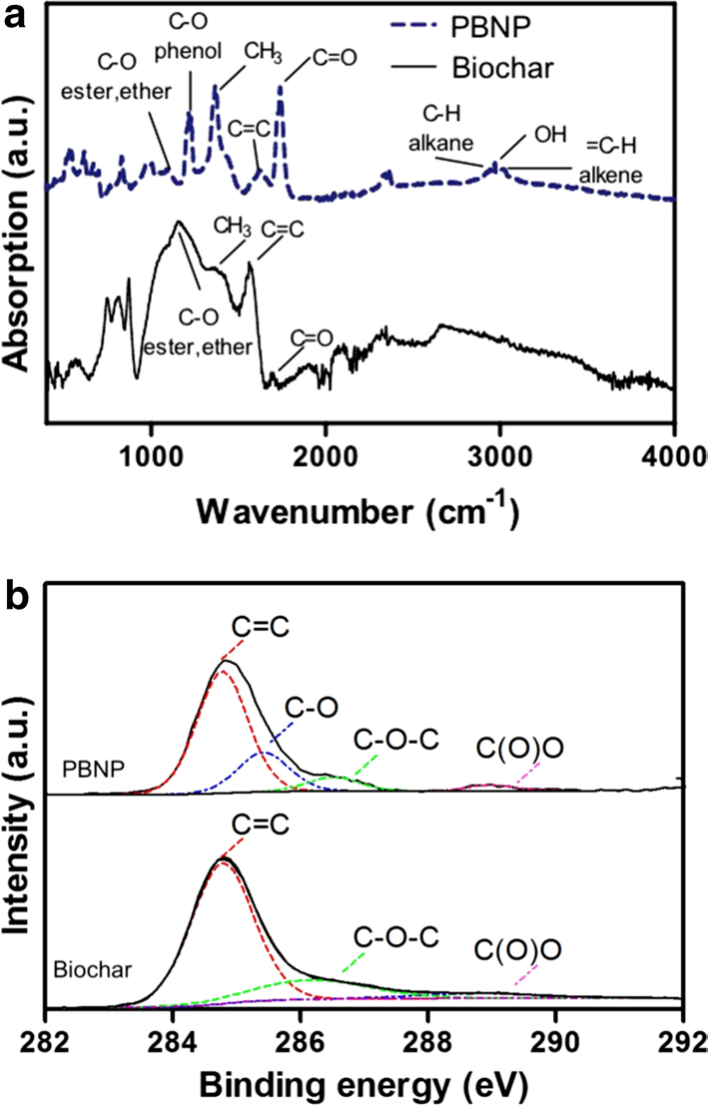
Fourier transform infrared (FTIR) spectra (**a**) and X-ray photoelectron spectroscopy (XPS) analysis (**b**) of bulk mixed wood chip biochar and the extracted BCNPs. (Adapted from Safari et al. (2019) with copyright permission from Springer Nature)

FTIR spectroscopy data show that, as compared to their parent biochar, PBNPs have higher abundances of C–O–C, aliphatic –OH, phenolic –OH, quinoid C=O (Liu et al. 2018; Goswami et al. 2022) and COOH groups (Song et al. 2019), which provides evidence that there are more oxygen containing functional groups on PBNPs than bulk biochar (Fig. 3). PBNPs also contain higher densities of functional groups than the bulk biochars from which they are derived, due to their higher surface area to mass ratios, suggesting that PBNPs may be more reactive in the environment and have higher contaminant sorption capacities. Zeta potential measurements also show that PBNPs are more negatively charged than bulk biochar due to the ionization of more –COOH functional groups, especially for PBNPs produced at low pyrolysis temperatures (Liu et al. 2018).

The physical properties of PBNPs, including shapes and sizes, vary among bulk biochars charred from various feedstocks, as shown in Table 1. Irregular, and sheet-like (Liu et al. 2018; Lian et al. 2018), spherical (Chen et al. 2017; Song et al. 2019) and carbon tube-like (Yi et al. 2015) shapes of PBNPs are reported in studies. The reported sizes of PBNPs range from 2.5 to 600 nm, which depend on the type of bulk biochar from which the PBNPs are derived. Interestingly, quantum-sized tiny particles with an average diameter of 2 nm were also observed (Song et al. 2019). Another distinct characteristic of PBNPs is their high specific surface area, owing to their nanoscale size. Existing data indicate a positive correlation between increasing pyrolysis temperature and elevated surface area. A review on bulk biochar from Ahmad et al. (2014) found that biochar produced > 400 °C was better suited for the removal of organic contaminants. The surface polarity and aromaticity are the most important surface characteristics for organic contaminant removal, whereas pH is the most important control for inorganic contaminants (Ahmad et al. 2014). Moreover, Xu et al. (2019) investigated the surface chemistry of PBNPs produced under different pyrolysis conditions from three different feedstocks and found that the biochar feedstock is the most influential factor on resulting PBNPs surface chemistry, followed by pyrolysis temperature. The authors noted that PBNPs yield varied between feedstocks, from 3.19% extracted from fruit tree branches to 34.26% from peanut straw at identical pyrolysis conditions. Thus, the biomass feedstock used has a profound impact on the generation and physicochemical properties of PBNPs, which may result in environmental impacts in various bulk biochar applications to soil and for water treatment. More studies need to be done to determine whether the trends identified in bulk biochar translate to PBNPs.

Our current understanding of the surface chemistry of naturally occurring pyrogenic carbon is limited. Santin et al. (2016) investigated the conditions of pyrolysis during a controlled forest fire in the Canadian boreal forest. The researchers found that temperatures during a forest fire primarily remain < 100 °C and only exceed 300 °C for a matter of minutes with a maximum temperature of ~ 745 °C (Santin et al. 2016). Naturally occurring pyrogenic carbon is important in the environment, and the production pyrolysis conditions are not like those of bulk biochar or associated PBNPs. Naturally occurring pyrogenic carbon, including PBNPs, and its surface chemistry is of interest because of the role that these particles may play in global elemental cycling now and in the geologic past.

### Influence on contaminant removal

#### Inorganic contaminants

Since PBNPs host appreciably higher densities of oxygen-bearing functional groups than their parent biochar, PBNPs have a high capacity to sorb inorganics, making them a potential replacement for costlier sorbents, such as activated carbon, in the remediation of wastewater contaminated with toxic heavy metal cations (Fu and Wang 2011). Much of PBNPs electron-donating capacity (EDC) is a result of the increased concentration of phenolic surface functional groups (Goswami et al. 2022). Qian et al. (2016) studied the removal of Cr(VI) and As(III) by PBNPs extracted by sonication of pyrolyzed rice straw. An inverse correlation between charring temperature and C, H and O contents of the PBNPs was observed at the studied pyrolysis temperatures of 100–700 °C, demonstrating a higher heavy metal removal capacity for PBNPs extracted from lower temperature biochar. They identified high EDC phenolic groups as those responsible for Cr(VI) reduction. Similarly, Dong et al. (2014) found that a higher content of polyphenolic organic compounds in PBNPs from Brazilian pepper biochar rendered them more effective in Cr(VI) reduction under acidic conditions due to the oxidation of phenolic compounds to carboxylic acid groups (Dong et al. 2014). FTIR spectroscopy analysis of these Cr(VI) laden biochar particles demonstrated a significant reduction in bands associated with hydroxyl groups while those of carboxyl groups increased, indicating PBNPs undergo oxidation at low pH according to reaction (1):

1 $${\text{HCrO}}_{{4}}^{ - } + {\text{ligands}} + {\text{7H}}^{ + } \to {\text{Cr}}^{{{3} + }} + {\text{partially oxidized ligands}} + {\text{CO}}_{{2}} + {\text{4H}}_{{2}} {\text{O}}$$

Interestingly, besides acting as a reducing agent, PBNPs can serve as an oxidant, similar to natural dissolved organic matter (Redman et al. 2002). The electron accepting capacity of PBNPs is commonly attributed to the presence of quinoid functional groups (Goswami et al. 2022). Dong et al. (2014) observed that oxidation of As(III), at an initial concentration of 10 mg L^−1^, increased from negligible to more than 20% at pH 10 by adding PBNPs in both ice and aqueous phases. Those authors ruled out the possibility of iron and manganese in colloids functioning as oxidizing species since characterization of mineral content in the PBNPs showed negligible amount of these metals. Nonetheless, inorganic species potentially released from PBNPs can both be sorbed and desorbed from the PBNPs surface. By using electron spin resonance spectroscopy, they identified semiquinone radicals as the primary electron accepting moiety since their concentration in the PBNPs decreased significantly after 24 h of As(III) oxidation. Dong et al. (2014) proposed that the oxidation reaction of As(III) can be enhanced under alkaline conditions, according to reaction (2):

2 $${\text{HAsO}}_{{2}} + {\text{3OH}}^{ - } + {\text{ligands}} \to {\text{H}}_{{2}} {\text{AsO}}_{{4}}^{ - } + {\text{reduced ligands}} + {\text{H}}_{{2}} {\text{O}}$$

FTIR spectroscopy characterization of As(III)-loaded PBNPs revealed a change in the position and intensity of peaks for carboxyl groups and semiquinone-bearing functional groups, and the appearance of a new As-O band. The effect of PBNPs on the simultaneous reduction of Cr(VI) and oxidation of As(III) was found to be limited to acidic and alkaline conditions, respectively, whereas under neutral conditions Cr(VI) reduction by As(III) was the dominant remediation mechanism. In a more recent study, Wei et al. (2018) extracted PBNPs from pyrolyzed Jerusalem artichoke stalk waste, a lignocellulose rich biomass, by mechanical shaking and filtration. Similar to the previous studies, they found that a higher charring temperature reduces the concentration of carboxyl and aliphatic carbons in PBNPs; however, quinonic and phenolic carbon contents remained relatively unchanged in the studied 300–700 °C pyrolysis temperature range. In addition, those authors studied the adsorption of copper ion to PBNPs by probing changes in the chemical structure of biochar derived DOM using Carbon K-edge near edge X-ray absorption fine structure (NEXAFS). The results showed that the carboxyl content of the studied PBNPs decreased as more copper, Cu(II), bound to their surface. Moreover, a higher binding affinity of Cu(II) to the colloids extracted from a higher temperature biochar was reported, although the causes underpinning the correlation between the copper binding affinity and pyrolysis temperature were not detailed (Wei et al. 2018). One possible reason for such a trend is a higher abundance of phenolic groups as compared to carboxyl groups at higher charring temperatures; the former are believed to possess a higher affinity for cations, as observed for bulk biochar (Pourret and Houben 2018). Nevertheless, a more systematic study is still required to shed light on affinity of individual functional groups towards metal ions.

While engineered biochar is produced for a specific application, sonication, shaking, and ball milling have been utilized to produce high surface area PBNPs from bulk biochar for metal cation adsorption (Peterson et al. 2012; Lyu et al. 2018a). Lyu et al. (2018a) optimized a ball milling procedure to maximize Ni(II) removal, and they found that increasing the ball-to-biochar ratio and milling time can improve Ni(II) uptake due to a significant increase in the surface area. Moreover, it was hypothesized that the mechanism of surface area enhancement varied depending on the pyrolysis temperature of the biomass: while ball milling increases the external surface area of low temperature carbonized biomass by reducing its grain size, it promotes collapse of internal pores in high temperature biochar and increases both external and internal surface areas. Similar to the previous studies, a noticeable increase in oxygen-bearing functional groups, and, therefore, the surface potential of ball milled biochar was evidenced by FTIR, Boehm titration, and zeta potential measurements. In terms of Ni(II) uptake, all ball milled biochars (PBNPs), regardless of their pyrolysis temperature and feedstock, demonstrated a significantly higher Ni(II) uptake, up to 650 µmol g^−1^, which testifies to the critical effect of increased external surface area on Ni(II) sorption. Moreover, three distinct phases were observed in the kinetics of Ni(II) sorption to PBNPs and bulk biochar: a relatively fast (< 30 min) initial Ni(II) diffusion across the water film in the biochar vicinity for both colloids and bulk biochars, an intra-particle diffusion phase which was significantly faster for PBNPs and identified to be the rate-limiting step in Ni(II) adsorption, and equilibrium with no measurable Ni(II) adsorption. The results highlight the effect of pore opening by ball milling , which accelerates the diffusion of Ni(II) into the particles. The underlying mechanisms of Ni(II) adsorption were proposed to be electrostatic attraction between enhanced oxygen-containing functional groups and Ni cations, and cation–π electron interactions due to the exposure of more of the graphitic structure of biochar by ball milling (Lyu et al. 2018a). Although complexation was also mentioned by Lyu et al. (2018a) as a possible governing adsorption mechanism, no further evidence was provided by the authors.

Similarly, in two recent studies, the governing mechanisms of lead sorption to PBNPs were studied and compared to their bulk biochar counterparts (Li et al. 2018; Cao et al. 2018). In both studies, precipitation of Pb(II) by anionic species released from the ash content of the biochars was found to be one of the main removal mechanisms for both bulk and PBNPs, as confirmed by XRD and FTIR analyses. Interestingly, the authors reported that the amount of ash content did not influence the removal of Pb(II). Instead, the amount of soluble species, such as carbonate and sulfate, determined the contribution of Pb(II) precipitation to overall removal, since most of the inorganic fraction consisted of SiO_2_ and Al_2_O_3_ (Li et al. 2018). Moreover, although PBNPs extracted by ball milling had a higher Pb(II) uptake as compared to that of the bulk biochar, the Pb(II) removal performance was not as significant as reported for Cu(II) or Ni(II) removal, perhaps due to different ball milling conditions and feedstock. Complexation of Pb(II) with functional groups was identified as the second most important removal mechanism in their earlier work (Li et al. 2018), while ion exchange was proposed as another major Pb(II) removal mechanism (Cao et al. 2018), although wheat straw was used as the biochar feedstock in both studies. The discrepancy between the proposed sorption mechanisms has not been explained by the authors, which demands further study.

Practical application of PBNPs as a bio-adsorbent may be limited because of their hydrodynamic size, typically 0.1–1.0 µm, which makes their separation from aqueous solution challenging. Wang et al. (2019) prepared 0.5 mm beads from ball milled bamboo biochar which were alginate crossed-linked by calcium cations. The resulting PBNPs showed Cd(II) uptake of > 2 mmol g^−1^, more than 5 times higher than that of PBNPs alone, perhaps because of the abundance of carboxyl groups on the alginate surface and exposure of its internal sorption sites upon mixing with PBNPs. However, the results of Cd(II) sorption kinetics experiments showed a chemical adsorption process which was significantly slower for the composite due to the slow diffusion of Cd(II) into alginate pores. Because of their size, such beads can be easily separated from treated water in a batch reactor or used in a fixed-bed operation (Wang et al. 2018a, b). In a recent study, Bai et al. (2020) prepared spheres of PBNPs by mixing them with mycelial (vegetative part of fungus) pellets. Cadmium uptake reached as high as 102 mg g^−1^ , for which surface complexation, cation exchange, and cadmium hydroxide precipitation were identified as the driving mechanisms.

Magnetic PBNPs have been proposed as a reusable nanosorbent in removing mercury and arsenic from aqueous systems (Nath et al. 2019; Li et al. 2020). Nath et al. (2019) showed more than 95% As(III) removal efficiency within 12 h at an As concentration of ≤ 500 ppb under neutral and alkaline pH range. The sorbent was regenerated by three different salt solutions including 0.5 NaCl, 0.5 M NaOH, and phosphate buffer saline. The As removal efficiency for all three regeneration solutions appeared to decrease after each regeneration cycle, a trend which has been reported by Li et al. (2020) for Hg(II) removal, although the decline in the Hg(II) removal capacity was less severe.

#### Organic contaminants

In addition to their metal cation retention characteristics, because of their carbonaceous structure and aromaticity, PBNPs have shown affinity towards hazardous organic contaminants which are found in industrial effluents, such as pharmaceutical products, dyes, herbicide, and polycyclic aromatic hydrocarbons (PAH) (Hasan and Jhung 2015; Yang et al. 2018; Safari et al. 2019; Yang et al. 2020a, b). For example, Tang et al. (2016) extracted PBNPs from various biochars by shaking and then determined their PAHs sorption coefficients. Hydrophobic interactions between the PAHs and PBNPs were found to be the driving mechanism of PAHs sorption, similar to natural dissolved organic matter (Tang et al. 2016). The sorption coefficients were found to be strongly dependent on the biochar source and the aromaticity of the resulting PBNPs. For example, colloids derived from pyrolyzed biomass, such as rice or soy-bean straws, showed positive correlations between their PAHs sorption coefficients and their degree of humification, as determined by specific ultraviolet absorbance measurements. The role of hydrophobic interactions in PAHs removal has been further corroborated in two recent studies (Fu et al. 2018; Hameed et al. 2020) where phenanthrene sorption to PBNPs was positively correlated to the pseudo-micellar conformation of the PBNPs and their aromaticity. Recently, a similar finding was reported on sorption of galaxolide, a synthetic musk widely used in cosmetics care products and detergents, to PBNPs derived from wheat straw and rice husk pyrolyzed at 300–700 °C (Zhang et al. 2019). A significant improvement in galaxolide uptake, from 330–746 to 609–2098 mg kg^−1^, was achieved by ball milling the biochar regardless of the pyrolysis temperature; however, the effects of ball milling on the mechanisms of galaxolide sorption were found to depend on the pyrolysis temperature. Ball milling increased only the external surface area of low temperature biochar, enhancing the surface adsorption. At higher pyrolysis temperatures (e.g., 700 °C) both internal and external surface areas along with pore volume and aromaticity increased, enhancing pore filling and π–π interaction removal mechanisms (Zhang et al. 2019).

Electrostatic interactions between PBNPs and organic pollutants, such as methylene blue or reactive red dyes, can also be promoted by mechanical treatment such as ball milling (Lyu et al. 2018a) or surface chemistry tuning such as doping (Xu et al. 2019). Lyu et al. (2018b) observed a significant increase in methylene blue uptake by ball milling biochars from different sources pyrolyzed at 300–600 °C. At pH < 3, below the isoelectric point of the studied PBNPs, PBNPs showed a higher uptake as compared to their parent biochar counterparts, primarily because of the increase in  surface area and subsequent enhancement of π–π interactions between the graphitic structures of PBNPs and aromatic methylene blue (Lyu et al. 2018b). Increasing the pH to 10 resulted in a significantly higher methylene blue uptake because of the deprotonation of carboxyl, lactonic, and phenolic hydroxyl groups, as reflected in the zeta potential of PBNPs. Such anionic groups can electrostatically adsorb cationic methylene blue and contribute to their overall uptake, while coexisting π–π interactions show less pH dependency.

**Fig. 4:**
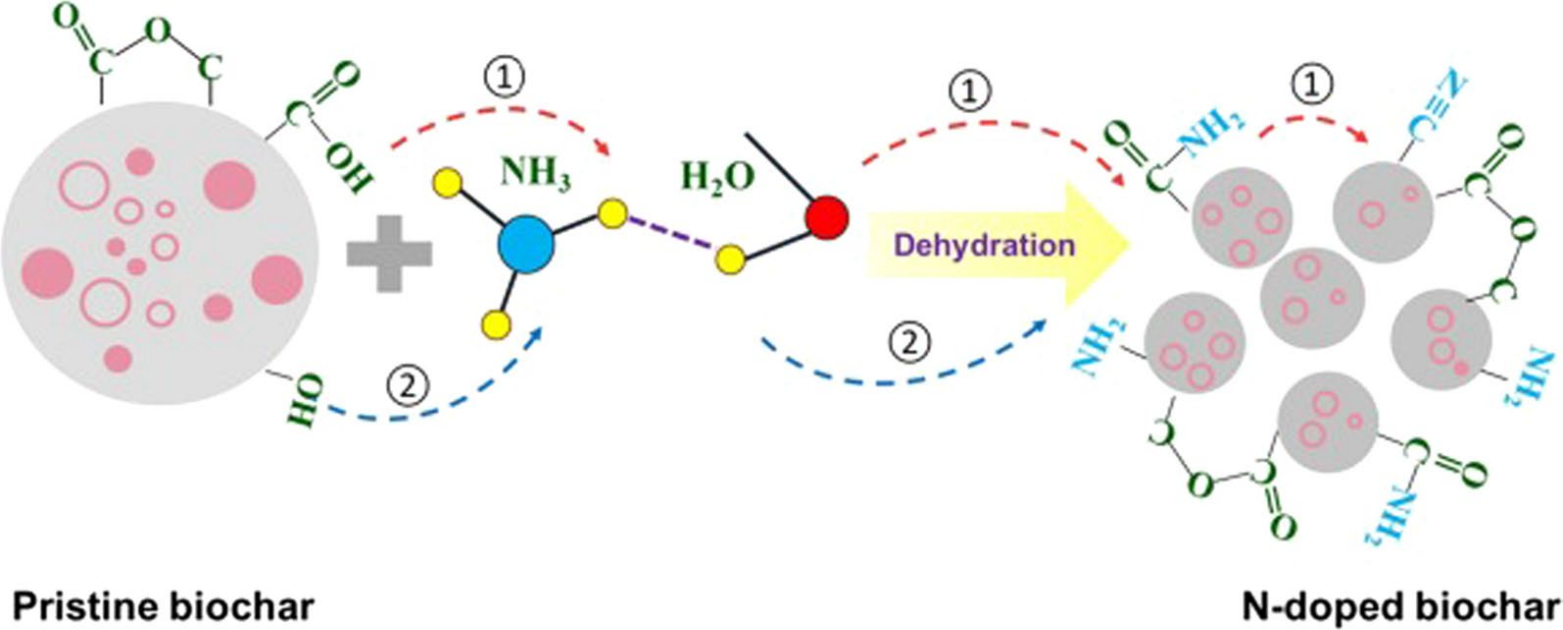
Mechanisms of doping nitrogen in biochar by ball milling in the presence of ammonium hydroxide. (Adapted from Xu et al. (2019) with copyright permission from Elsevier)

Doping PBNPs with nitrogen through the ball milling of bulk biochar with ammonium hydroxide imparts the resulting particles with functional groups which have net positive charge at their protonated state, as evidenced by X-ray photoelectron spectroscopy (XPS), FTIR, and zeta potential measurements (Xu et al. 2019). The N species are believed to be generated during dehydration of carboxyl and phenolic hydroxyl groups which have higher densities in biochar pyrolyzed at lower temperatures (Fig. 4). Such positively charged binding sites favor electrostatic sorption of anionic reactive red dye, a model organic pollutant, and increase its uptake by 2–4 times as compared to pristine PBNPs, depending on the starting biomass and pyrolysis temperature.

While pristine biochar can be separated and reused after organic pollutants removal, separation of PBNPs from treated water and their reuse at an industrial scale can be challenging. Coupling PBNPs with magnetite nanoparticles has been shown to be a viable approach to address this issue since the resulting magnetic composite can be collected, regenerated and reused (Shan et al. 2016; Dong et al. 2018). Shan et al. (2016) studied sorption of two pharmaceutical compounds, carbamazepine and tetracycline, to a ball milled mixture of biochar and Fe_3_O_4_. Longer ball milling increased the specific surface area of the admixture, leading to a higher removal of organic compounds in water treatment experiments. The spent magnetic sorbent was collected by a magnet and the sorbed organic compounds were degraded to smaller molecules by ball milling (Shan et al. 2016). Dong et al. (2018) used a coprecipitation method to deposit magnetite on PBNPs, a combination of grinding biochar and filtration, and investigated their potential as a reusable bioadsorbent for 17β-estradiol. The magnetic PBNPs presented a similar performance to the original PBNPs, and they maintained their 17β-estradiol uptake capacity after five cycles of regeneration by ozonation.

In a different approach, Wang et al. (2019) prepared a composite of ball milled biochar with calcium alginate (CA-BMB) and studied its methylene blue adsorption capacity and kinetics. Through adsorption isotherm experiments, the resulting methylene blue uptake was determined to be 1210 mg g^−1^ for CA-BMB, slightly lower than that of pristine calcium alginate beads, but significantly higher than that of the ball-milled biochar alone. By compositing BCNPs with TiO_2_ nanoparticles, Safari et al. (2019) demonstrated that the PBNPs can not only sorb methylene blue through electrostatic attraction but also enhance the demethylation efficiency of the TiO_2_ photocatalysts, reportedly by increasing density of negative charge and methylene blue. Use of PBNPs as a photocatalyst promoter deserves further studies to unravel their precise contribution to photocatalysis efficiency.

## Discussion and future research directions

### Pyrolyzed biomass-derived nanoparticles influence on contaminant mobility

A thorough investigation of PBNP mobility in the subsurface is necessary for a complete understanding of their fate in natural environments beyond adsorption and degradation of contaminants. Contaminant sorption, reduction, oxidation, and degradation experiments conducted in the laboratory provide a framework for potential field-scale applications and scalable industrial processes. Experiments conducted under specific chemical conditions need to consider the numerous environmental factors that influence not only contaminant speciation and reactivity, but also the reactivity and physiochemical properties of the adsorbents as well. These environmental parameters include, but are not limited to, pH, ionic strength, sorbent–sorbate ratio, biological reactions occurring, organics present (e.g., humic and fulvic acids), and various competing reactive surfaces present in natural systems (e.g., bacterial cells, clay minerals, metal oxyhydroxides).

Recent research has added to our knowledge of the mobility of AC colloids in the environment. For example, following a successful demonstration of the dechloronation of PCE and TCE by CICs as well as by PBNPs (Bleyl et al. 2012), Busch et al. (2014) conducted column experiments to determine CIC mobility in quartz sand, glass beads, and aquifer samples from a proposed CIC field-test site. The researchers found a negative correlation between ionic strength and CIC mobility (Busch et al. 2014). An increase in ionic strength, with the highest studied value of 200 mM CaCl_2_, resulted in nearly complete inhibition of CIC mobility. Moreover, a positive correlation was identified between pH and colloid mobility. At a pH of < 4, there was complete inhibition of CIC mobility, whereas gradually increasing pH gradually increased mobility (Busch et al. 2014). Experimental studies, such as the one described here, provide valuable insights into the feasibility and effectiveness of biomass-derived nanoparticles prior to large-scale nanoremediation projects. Moreover, Georgi et al. (2015) found that CICs demonstrated increased mobility in the presence of humic acids and cellulose during water column experiments.

Studies on the chemistry of biochar-derived PBNPs have also begun to constrain factors controlling their mobility in natural environments. Recent studies show that the mobilization of PBNPs in lower ionic strength paddy soils is significant, which indicates that PBNPs in soil can be transported into groundwater (Chen et al. 2017). In a separate study, Wang and coworkers (2013) showed that PBNPs have a high mobility in water-saturated quartz sand, indicating that they are likely to be mobile in alluvial aquifers. Biochar has also been added to agricultural fields containing organic compounds such as humic and fulvic acids, and proteins from animal manure (Yang et al. 2019a, b; Wang et al. 2020a, b). It was demonstrated that, together with ionic strength and pH, such organic matter can influence the release of PBNPs from bulk biochar. At 1 mM NaCl and under acidic conditions, the release of PBNPs was increased and decreased by the presence of humic substances and proteins due to electrostatic repulsion and attraction, respectively. Higher pH or ionic strength diminished the effect of humic acids, which was attributed to the strong buffering capacity of the PBNPs. Thus, metals and organic contaminants that are immobilized on PBNPs in the environment are likely to be transported with PBNPs and may ultimately have detrimental impacts to the ecosystem depending on the water chemistry and geologic medium (Jin et al. 2020; Liu et al. 2020).

### Photochemistry of PBNPs: a dissolved black carbon (DBC) pool

Since PBNPs comprise a large fraction of the dissolved black carbon (DBC) pool, we review the photochemistry of DBC released from bulk biochars in this section. Fu et al. (2016) showed that DBC released from biochar can generate reactive oxygen species. During that process, carbonyl-containing structures on DBC are involved in the sensitization of singlet oxygens. The generation of superoxide species is believed to depend on electron transfer reactions mediated by silica minerals in DBC, while the phenolic structures serve as the electron donors. ROS generated by dissolved organic matter (DOM) are known to mediate the indirect photolysis of many organic contaminants as well as the redox reactions of metals (Nico et al. 2002; Lam et al. 2003). In a detailed study, Fang et al. (2017) investigated the photodegradation of diethyl phthalate, as a model organic contaminant, in the presence of biochar, and quantified the individual contributions of the bulk biochar matrix and the PBNPs to the photogeneration of reactive oxygen species. The particles extracted by sonication contributed significantly to singlet oxygen (^1^O_2_) generation under UV illumination, whereas their contribution to •OH generation was minimal. The photogeneration of the aforementioned species depended on the pyrolysis temperature of the biochar, which also has a direct influence on the structure of the resulting colloids and their ability to transfer electrons to O_2_ molecules and generate ^1^O_2_ (Fu et al. 2016; Fang et al. 2017). In a more recent publication, the apparent quantum yield of biochar nanoparticles, produced by sonication, was compared against those of four model humic acids using 17β-estradiol as a model pollutant (Zhou et al. 2018). While •OH photo-generation by the biochar nanoparticles was found to be insignificant, photo-formation of the triplet excited state biochar, which acted as electron acceptor in the presence of 17β-estradiol, was reported to be the dominant mechanism in degrading the estradiol. The electron transfer from 17β-estradiol to PBNPs causes their phenolic hydroxyl groups to become radical, promoting the self-coupling and formation of the 17β-estradiol dimer, trimer, or oligomer (Zhou et al. 2018). Smaller molecular size and a higher extent of aromaticity of PBNPs were identified as primary reasons for the superior photo-transformation activity of the PBNPs as compared to humic acids. PBNPs may have unique or novel nanoscale-dependent properties due to their size and abundance of oxygen-containing functional groups. Thus, we hypothesize that PBNPs are likely to be efficient catalysts of photochemical reactions in the environment, impacting the fate of metals and organic contaminants. However, our current knowledge on photochemical redox reactions of metals and the photolysis of organic contaminants induced by PBNP is lacking and needs further investigation.

### Gaps in current understanding

Given the abundance of studies presently being conducted with ENPs, it is clear that PBNPs remain understudied. Much of the literature is focused on carbon-based ENPs such as graphene oxides, carbon nanotubes, and fullerenes (Maitlo et al. 2019; Lal et al. 2020). Meanwhile, little attention has been afforded to the leaching of PBNPs from AC environmental remediation applications, the potential leaching of PBNPs from bulk biochar applications for agriculture, and the environmental impacts of the use of novel PBNPs applications in contaminant removal. The study of PBNPs, whether derived from AC or biochar, should first aim to understand the types of biomass and pyrolysis conditions that produce bulk carbon materials that generate PBNPs in the environment. Further, studies of PBNPs should employ spectroscopic techniques to understand the specific mechanisms of contaminant binding on the nanoparticles and use flexible and predictive adsorption modeling approaches that can incorporate knowledge gained from spectroscopic analyses, such as surface complexation models. This mechanistic knowledge of the surface chemistry and reactivity of PBNPs is needed to develop accurate models of nanoparticle transport and contaminant delivery, to inform PBNP mobility in the environment.

In addition to further understanding of contaminant interactions and mobility in the environment, a more complete knowledge of the fate of PBNPs in nature is necessary prior to wide-spread utilization. There is a paucity of literature on the risks of introducing PBNPs to the environment either intentionally or through leaching of the parent material. Recently, Vogel et al. (2018) investigated the influence of CICs on PCE dechloronation and simultaneous microorganism stimulation in the subsurface, specifically *Polaromonas* sp., an organohalide-respiring bacterium. Weil et al. (2019) investigated the effect of CICs on the toxicity of various organisms, including the commonly studied crustacean *Daphnia magna*, algae (*Scenedesmus vacuolatus*), and the insect *Chironomus riparius* in the same field site in Germany as described above by Mackenzie et al. (2016). The studies mentioned identified that the environmental benefit, in this case the degradation of TCE, outweighed potential ecological harms, such as the shading of aquatic systems resulting in decreased photosynthesis in algae (Weil et al. 2019). These investigations, which build on previous site data, provide a multifaceted approach to understanding the long-term and ecological effects of colloidal carbon (e.g., CIC) applications. When considering that PBNPs result from leaching of bulk AC or biochar applications, there is considerable risk for ongoing and unknown ecological impacts.

The above studies provide examples of the groundwork necessary to define the precautions needed to progress nanoremediation via PBNPs. An area of concern when considering PBNPs in the environment is changes in ecotoxicity as well as physical and chemical properties with varying scale. This complexity can be observed in Table 3, demonstrating a threefold variation in PBNP size and a wide variety of structures, ranging from spherical to sheet like. These contrasting sizes and structures can influence contaminant affinity, mobility, and biological uptake. The contrasting properties between macro- and nano-scale materials have resulted in proposals for separate regulatory frameworks (Kookana et al. 2014). There are recent investigations into aging and weathering effects on bulk biochar applications (Zhong et al. 2020), but there is a need for increased focus on the long-term effects of PBNPs and their lifecycle following release from bulk applications. These should include real-world sampling for PBNPs downstream from previous bulk applications as well as testing of aquatic organisms. Such studies promise to shed light on the persistence and longevity of the PBNPs as well as their role in the fate of contaminants in the environment.

**Table 3:** Physical characterization of bulk biochar and biochar nanoparticles (BCNPs)

Samples^a^	Nanoscale^b^ (nm)	Surface area^c^ (m^2^ g^−1^)	Structure	References
N-PS-500	50–500^d^, 30–200^e^	–	Irregular^d^, Nanotube^f^	Yi et al. (2015)
N-WC-500	2.5–5.5	–	Ipherical	Chen et al. (2017)
N-PS-300	13.2–21.6	63.60	Irregular	Liu et al. (2018)
N-PS-600	13.4–37.2	264.00	Irregular	Liu et al. (2018)
N-RS-400	30–600	93.18	Irregular sheet-like	Lian et al. (2018)
N-RS-700	30–600	253.90	Irregular sheet-like	Lian et al. (2018)
N-SS-500	50	–	Spherical	Song et al. (2019)
N-WS-600	700–1000	–	Irregular	Yang et al. (2019a, b)
N-WS-600	200–400	–	Irregular	Wang et al. (2020a, b)

^a^N-XXX-T (N represents BCNPs; PS: peanut shell biochar, WC: wood chip biochar, RS: rice straw biochar, SS: sewage sludge biochar, WS: wheat straw; T represents pyrolytic temperature.)

^b^Data was obtained from Transmission Electron Microscopy (TEM)

^c^Surface area was calculated by the Brunauer–Emmett–Teller (BET) model

^d^Hard BCNPs

^e^Soft BCNPs

^f^Appearing in a trace amount

The comprehensive laboratory research being conducted on PBNPs derived from biochar continues to build our understanding of the surface reactivity of PBNPs. These efforts are supporting accurate predictions of the fate of contaminants in the environment in the presence of PBNPs. As applications of bulk biochar have been employed in recent years and are expected to continue, this is a crucial step in understanding the long-term effects of derived PBNPs on contaminant fate in the environment and ecotoxicology of the PBNPs themselves. Unfortunately, PBNPs derived from AC have received considerably less attention and there remain significant gaps in understanding their reactivity in comparison to the parent material. These gaps in understanding present risks, but also considerable research potential to increase our knowledge of the long-term effects of bulk AC applications.

## Data Availability

Data sharing not applicable to this article as no datasets were generated or analysed during the current study.
